# IFITM1 expression is crucial to *gammaherpesvirus* infection, *in vivo*

**DOI:** 10.1038/s41598-018-32350-0

**Published:** 2018-09-20

**Authors:** Hosni A. M. Hussein, Katarina Briestenska, Jela Mistrikova, Shaw M. Akula

**Affiliations:** 1 Department of Microbiology & Immunology, Brody School of Medicine at East Carolina University, Greenville, NC 27834 USA; 2 Department of Microbiology and Virology, Faculty of Natural Sciences, Comenius University in Bratislava, Mlynská dolina, SK-842 15, Bratislava, Slovak Republic; 3 Institute of Virology, Biomedical research Center, Slovak Academy of Sciences, Dubravska cesta 9, 845 05, Bratislava, Slovak Republic

## Abstract

The oncogenic *gammaherpesviruses*, Epstein–Barr virus (EBV) and Kaposi’s sarcoma herpesvirus (KSHV), are etiologically associated with a variety of human cancers, including Burkitt’s lymphoma (BL), Hodgkin lymphoma (HL), Kaposi’s sarcoma (KS), and primary effusion lymphoma (PEL). Recently, we demonstrated KSHV infection of B- and endothelial cells to significantly upregulate the expression of interferon induced transmembrane protein 1 (IFITM1) which in turn enhances virus entry. This is an extension of the above study. In here, we determined EBV infection of cells to trigger IFITM1 expression, *in vitro*. Silencing IFITM1 expression using siRNA specifically lowered *gammaherpesvirus* infection of cells at a post binding stage of entry. A natural model system to explore the effect of IFITM1 on *gammaherpesvirus* infection *in vivo* is infection of BALB/c mice with murine *gammaherpesvirus* 68 (MHV-68). Priming mice with siRNA specific to IFITM1 significantly lowered MHV-68 titers in the lung specimens compared to priming with (NS)siRNA or PBS. MHV-68 titers were monitored by plaque assay and qPCR. Taken together, for the first time, this study provides insight into the critical role of IFITM1 to promoting *in vivo gammaherpesvirus* infections.

## Introduction

Kaposi’s sarcoma-associated herpesvirus (KSHV) is the etiological agent of Kaposi’s sarcoma (KS), the most common cancer afflicting HIV-infected individuals^1^. KS is characterized by three histological features: angiogenesis, inflammation, and proliferation^2^. KSHV is also associated with two other B cell lymphoproliferative disorders: primary effusion lymphoma (PEL) and multicentric Castlesman disease (MCD)^3^. KSHV belongs to the *γ-herpesvirinae* subfamily (genus *Rhadinovirus*) and was first described in 1994^4,5^.

KSHV internalization is a highly orchestrated but complicated event yet to be thoroughly understood. The initial step in the virus entry process is the reversible step of binding or attachment to the target cells^6^. This step is primarily achieved by KSHV interacting with the ubiquitously expressed cell surface receptor, heparan sulfate (HS)^6,7^. The attachment to cells enables KSHV to interact with entry promoting receptors allowing the virus to eventually enter the cell^8,9^. In a recent study, we determined that interferon (IFN)-induced transmembrane-1 (IFITM1) expression to significantly enhance KSHV infection of human B and endothelial cells^10^. In opposition to this process, over-expression of microRNA (miRNA)-36 (miR-36) significantly lowered expression of IFITM1 and thus the virus entry. We concluded IFITM1 to play a crucial role in promoting early stages of KSHV infection of cells, *in vitro*.

Over the past few years, several novel genes downstream of type I IFN signaling that inhibit infection by individual or multiple families of viruses have been described. There are two genetically and functionally distinct families of interferon stimulated genes (ISGs) that have antiviral properties, they are IFN-induced proteins with tetratricopeptide repeats (IFIT) and IFITMs. IFITs contribute to an antiviral state against some viruses by binding components of the eIF3 translation initiation complex and inhibiting protein translation^11^. In contrast, IFITMs block viral infection at the entry stage^12^. IFITMs are a double-edged sword when it comes to influencing viral entry; they can also enhance viral entry^13,14^. Interestingly, IFITM1 expression not only enhanced KSHV infection of cells, but also infection of cells with a closely related herpesvirus belonging to the *γ-herpesvirinae* subfamily, Epstein-Barr virus (EBV)^10^.

Both KSHV and EBV are the most relevant human *γ-herpesviruses*. Therefore, we conducted further studies to confirm the role of IFITM1 in KSHV and EBV infections, *in vitro* and *in vivo*. For *in vivo* studies, we used murine γ-herpesvirus 68 (MHV-68), that serves as a good model to understand *γ-herpesvirus* (KSHV and EBV) pathogenesis^15,16^. Herein, we provide pioneering evidence to demonstrate a key role for IFITM1 in the *in vitro* and *in vivo* infection of *γ-herpesviruses*.

## Results

### Infection of BJAB cells wit *γ-herpesviruses* induce expression of IFITM1

**Figure 1:**
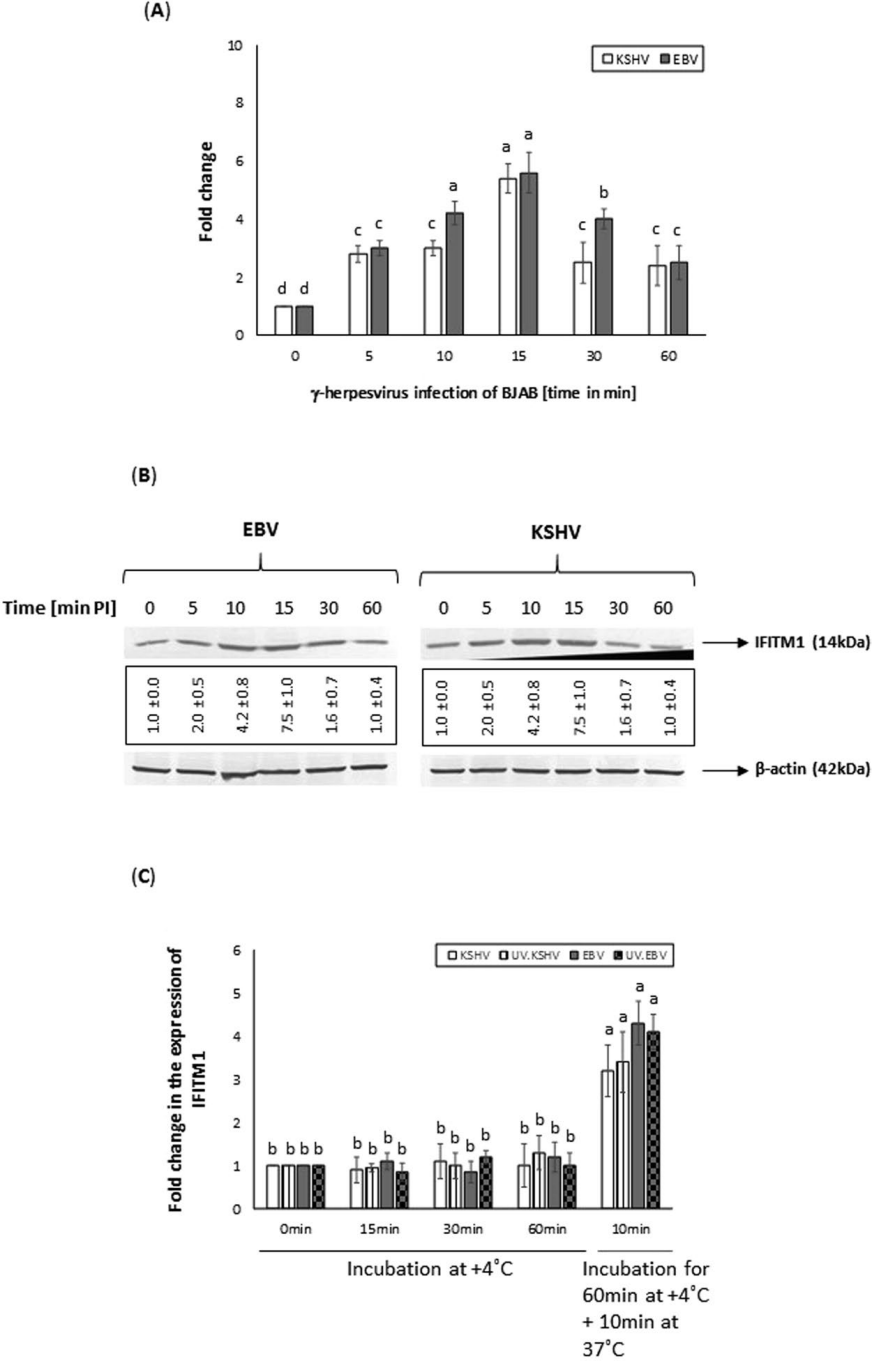
Infection of BJAB cells with EBV and KSHV induce expression of IFITM1. (**A**) The relative expression of IFITM1 in EBV or KSHV infected BJAB cell was monitored by qRT-PCR. The expression was measured in terms of cycle threshold value (Ct) and normalized to expression of β-actin. The *x-axis* denotes the time point post virus infection in minutes and the *y-axis* denotes fold change in expression of IFITM1. (**B**) Western blotting analysis demonstrates EBV or KSHV infection of BJAB cells to increase IFITM1 protein levels. Expression of IFITM1 levels was normalized to β-actin protein levels. Data representing the IFITM1 protein expression levels are presented as fold increase (average ± s.d. from three experiments) in the boxes below the panels. (**C**) Virus binding to cells is not sufficient to induce IFITM1 expression. BJAB cells were incubated with 10 MOI of wild-type and UV inactivated viruses for different time points at +4 °C or 60 min at +4 °C plus a 10 min incubation at 37 °C prior to monitoring expression of IFITM1 by qRT-PCR. Bars **(A**,**C)** represent average ± s.d. of five individual experiments. Columns with different alphabets indicate the values to be statistically significant (p < 0.05) by least significance difference (LSD). The Western blot results (**B**) presented are a representative data and the original full-length blots for EBV and KSHV of the cropped images is provided in Supplemental Figs 3 and 4, respectively.

In a recently concluded study, we demonstrated the ability of KSHV to induce IFITM1 expression during early stages of infection^10^. In the present study, we analyzed the effect of another closely related *γ-herpesvirus*, EBV, on IFITM1 expression. IFITM1 transcript (Fig. 1A) and protein expression (Fig. 1B) levels were significantly elevated with EBV and KSHV infection of BJAB cells. The expression of IFITM1 increased in virus infected cells as early as 5 min post infection (PI) which was elevated by 10 min and 15 min PI in BJAB, but significantly declined by 30 min PI (Fig. 1B). We observed a similar IFITM1 expression profile during early stages of viral infection in human microvascular dermal endothelial cells (HMVEC-d) cells (Supplemental Fig. 1). We also performed binding assays to decipher if just binding of KSHV and EBV is enough to induce IFITM1 expression in BJAB cells. Our results (Fig. 1C) demonstrate binding of the wild-type or the UV.inactivated virus is not enough to induce IFITM1 expression. The results implicate the ability of *γ-herpesviruses* to induce the expression of IFITM1 during early stages of infection.

### IFITM1 expression is a necessity for *γ-herpesvirus* infection of cells

**Figure 2:**
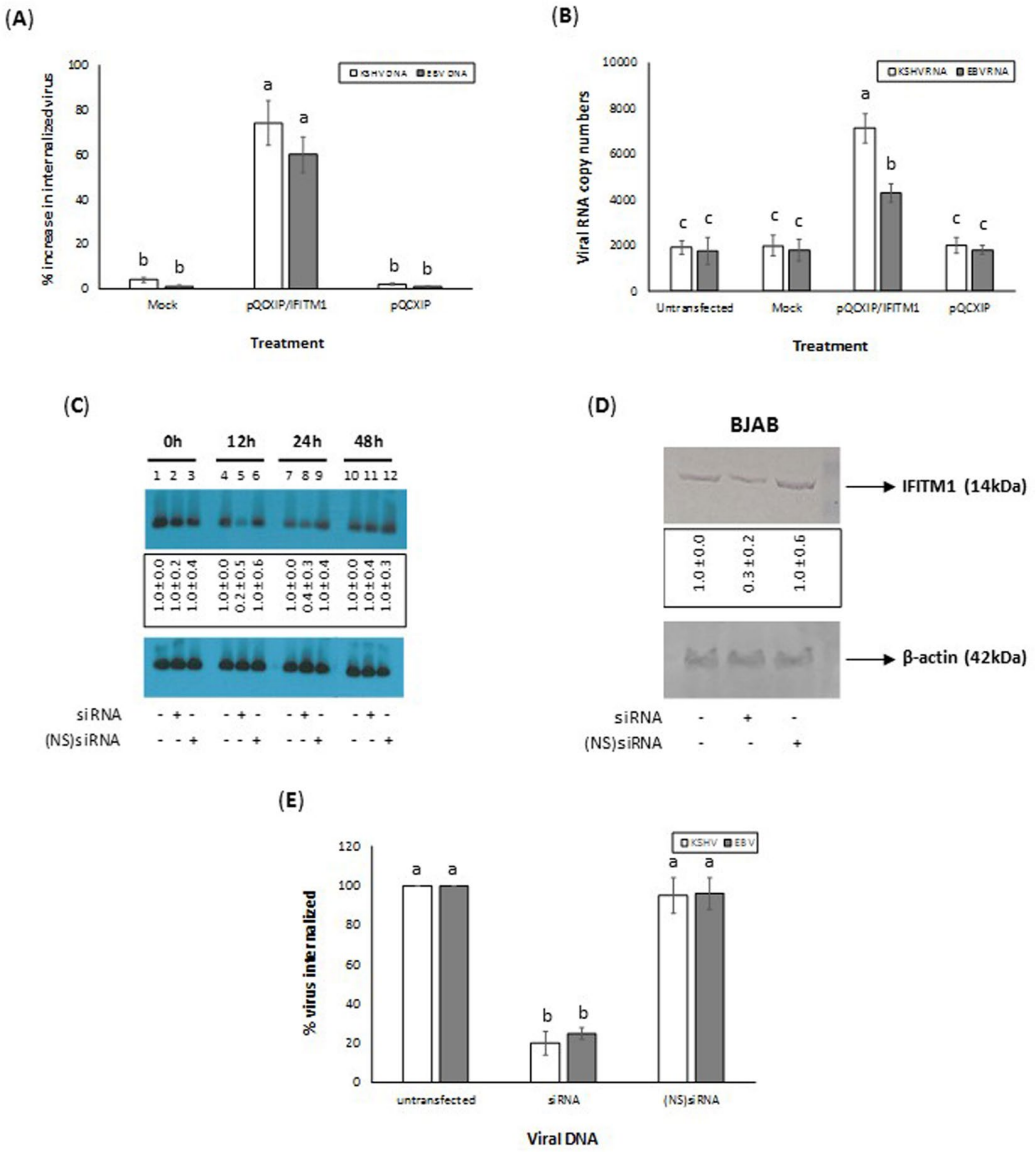
IFITM1 expression is a necessity for EBV and KSHV infection of cells. Overexpression of IFITM1 enhances EBV and KSHV infection of cells. BJAB cells were untransfected, mock transfected, transiently transfected with pQCXIP/IFITM1, or pQCXIP prior to infecting with 10 MOI of EBV or KSHV. Data was plotted to represent the percentage increase in the virus infection of different cells by monitoring (**A**) the copy numbers of internalized viral DNA in different cells compared to untransfected cells or (**B**) change in RNA copy numbers of *BRLF1* and *ORF50* of EBV and KSHV, respectively. (**C**) Northern blotting to monitor the effect of transfecting cells with siRNA specific to IFITM1. Target cells were untransfected or transfected either with ds siRNA or (NS)siRNA controls. After 0, 12, 24, and 48 hours after transfection, total RNA was isolated from the cells and subjected to Northern blotting to monitor IFITM1and β-actin mRNA. The results presented are a representative data and the original full-length blots of the cropped images is provided in Supplemental Fig. 4. (**D**) Western blot demonstrating the effect of silencing the expression of IFITM1 using siRNA. Cells transfected with siRNA specific to IFITM1 were lysed 12 h post transfection. The cell lysates were resolved on a SDS-PAGE gels, proteins transferred on to a PVDF membrane prior to conducting Western blotting using appropriate antibodies. The results presented are a representative data and the original full-length blots of the cropped images is provided in Supplemental Fig. 5. Data representing the IFITM1 (C) mRNA and (**D**) protein expression levels are presented as fold increase (average ± s.d. from three experiments) in the boxes below the panels. (**E**) Silencing the expression of IFITM1 by siRNA significantly decreased EBV and KSHV infection of cells. BJAB cells were either un-transfected, transfected with IFITM1-specific siRNA, or transfected with (NS) siRNA before infecting with KSHV, or EBV. Data was plotted to represent the percentage of virus infection as determined by monitoring the change in copy numbers of internalized viral DNA. Bars (**A**,**B**,**E**) represent average ± s.d. of five individual experiments. Columns with different alphabets indicate the values to be statistically significant (p < 0.05) by LSD.

In a recently concluded study, we demonstrated a crucial role for IFITM1 expression in KSHV infection of cells. This was possible by monitoring the expression of *ORF50* transcript as a measure of infection. In the current study, we analyzed internalization of the *γ-herpesviruses* by monitoring the internalized viral DNA (Fig. 2A) compared to the expression of *ORF50* and *BRLF-1* transcripts (Fig. 2B). BJAB cells expressing IFITM1 supported a significantly enhanced KSHV and EBV infection compared to those cells that were left untransfected, mock transfected, or transfected with the empty vector. To authenticate the role for IFITM1 in enhancing infection of *γ-herpesviruses*, we tested the effect of silencing IFITM1 on the internalization of the viruses. Briefly, we first transfected cells with siRNA specific for IFITM1. Northern blotting and Western blotting were performed at 0, 12, 24, and 48 hours after transfection as per the standard protocols to monitor IFITM1 mRNA and protein expression levels, respectively. A maximum IFITM1 mRNA (Fig. 2C) and protein (Fig. 2D) inhibition were observed in cells transfected with siRNA specific to IFITM1 at 12 h post transfection. On the same lines, internalized *γ-herpesviruses* in cells silenced for the expression of IFITM1 was significantly lower compared to cells that were untransfected or transfected with (NS)siRNA (Fig. 2E). Taken together, the results clearly implicate a role for IFITM1 in enhancing KSHV, and EBV infection of cells.

### IFITM1 enhances virus infection at a post-attachment stage

**Figure 3:**
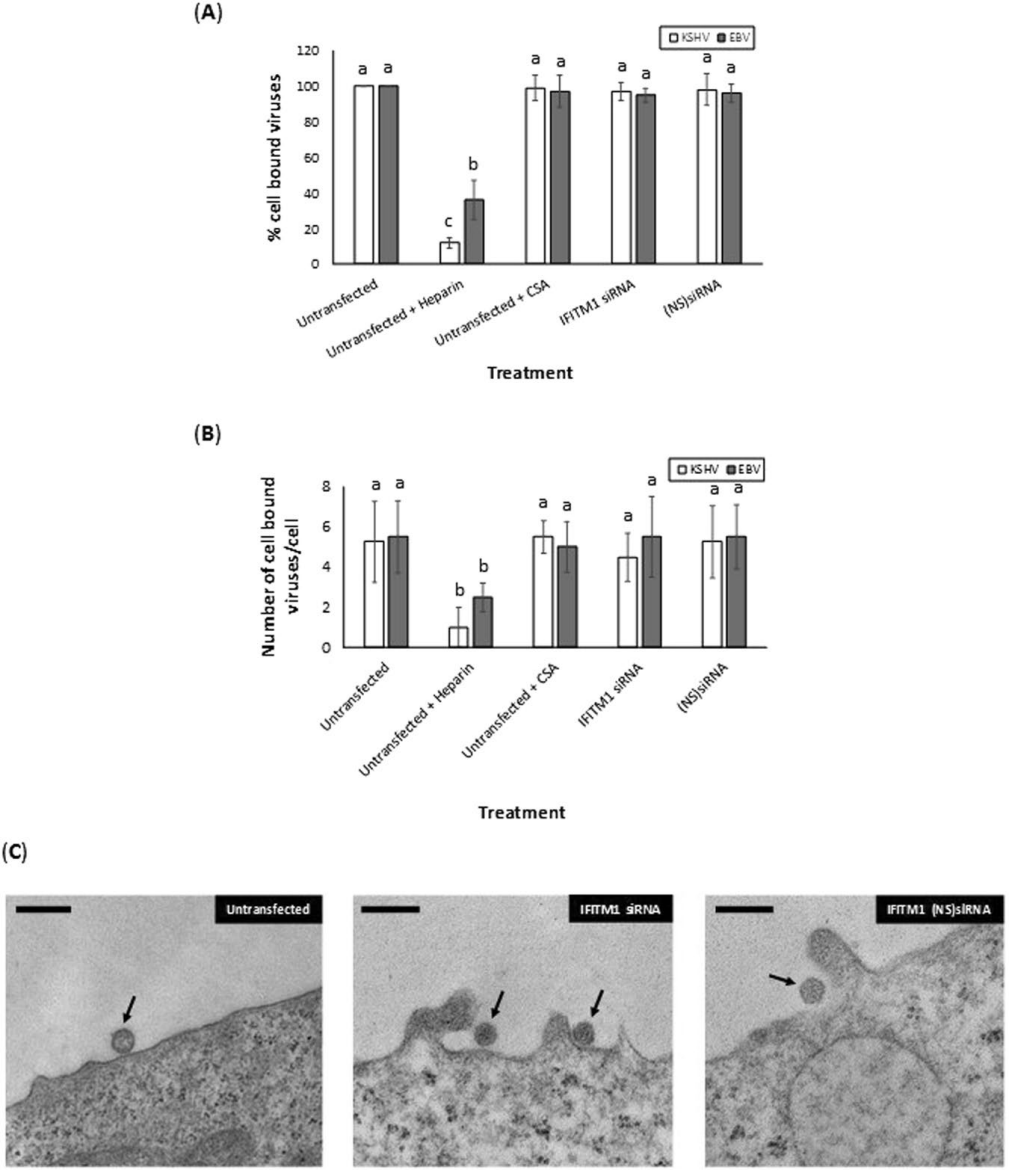
IFITM1 enhancement of EBV and KSHV infection of cells is at a post-attachment stage of virus entry. (**A**) EBV and KSHV binding to BJAB cells were monitored in cells that were untransfected, untransfected and treated with heparin or CSA, or transiently transfected with IFITM1-specific siRNA, or transfected with non-specific (NS) siRNA. Data was plotted to represent the percentage of EBV or KSHV binding to BJAB cells treated differently compared to the untransfected cells. (**B**) EBV and KSHV were allowed to bind at 4 °C to BJAB cells that were untransfected, or transiently transfected with IFITM1-specific siRNA, or (NS) siRNA. Viruses incubated with soluble heparin and chondroitin sulfate served as controls. After 60 min, cells were fixed with 2% glutaraldehyde. Thin sections were examined by transmission electron microscopy. Enveloped virus particles bound to the cell surfaces of randomly selected 10 cells were counted by three different individuals using three different grids of their choice and the numbers were averaged. (**C**) Representative electron micrographs of KSHV binding to BJAB cells is depicted. Enveloped virus particles bound to the BJAB cells surfaces are indicated by the arrows. Magnification: 82,000 × (solid bar denotes 500 nm). Bars **(A)** represent average ± s.d. of five individual experiments. Columns (**A**,**B**) with different alphabets indicate the values to be statistically significant (p < 0.05) by LSD.

To enumerate the role of IFITM1 on binding of viruses to target cells, we used untransfected or BJAB cells transfected with siRNA to IFITM1, or (NS)siRNA. These cells were used in binding assays. The binding assay performed on BJAB cells demonstrated that the transfection of cells with (NS)siRNA or siRNA specific to IFITM1 did not block 10 multiplicity of infection (MOI) of KSHV and EBV from binding the target cells (Fig. 3A). Incubating KSHV with heparin but not CSA significantly blocked KSHV and EBV from binding cells (Fig. 3A). We observed identical results when 1 MOI (Supplemental Fig. 6) and 0.1 MOI (data not shown) of KSHV and EBV were used in the above binding assays. This data was confirmed by electron microscopy by monitoring KSHV **(**Fig. 3B,C**)** and EBV (Fig. 3B) binding to BJAB cells. Based on these results, we concluded IFITM1 to alter virus infection at a post-attachment stage of entry.

### Silencing IFITM1 expression in BALB/c mice lowered MHV-68 infection

**Figure 4:**
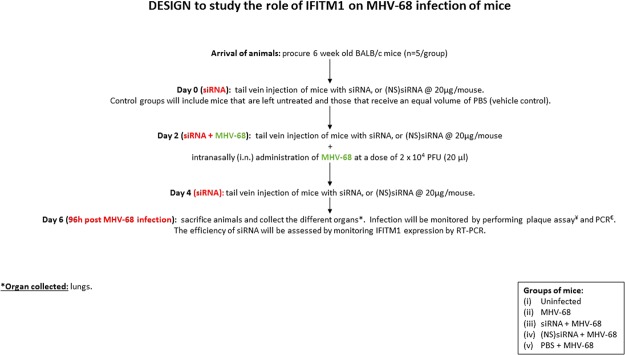
Schematic depicting the animal study design. The study was approved by the State Veterinary and Food Administration of the Slovak Republic (2937/10221) and strictly followed the European Union standards.

**Figure 5:**
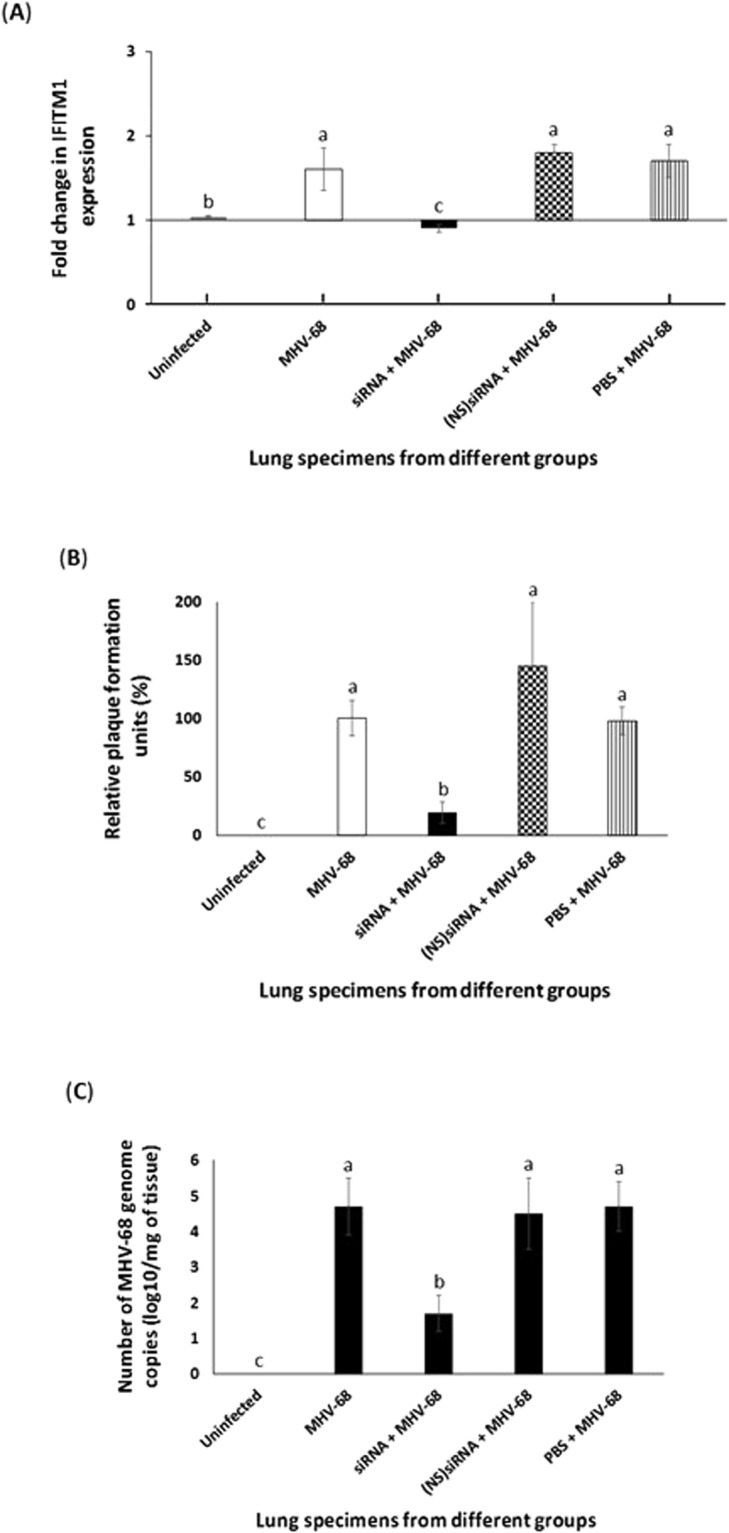
Silencing IFITM1 expression in BALB/c mice lowered MHV-68 infection. The four groups of mice (n = 5/group) that were used in this study are as follows: (i) uninfected; (ii) MHV-68 infected; (iii) IFITM1 siRNA primed + MHV-68 infected; (iv) (NS)siRNA primed + MHV-68 infected; and (v) PBS primed + MHV-68 infected. On 7dPI, expression of (**A**) IFITM1 or (**B**,**C**) virus titer by (**B**) plaque assay and (**C**) qPCR was determined. (**B**) Replication of the MHV-68 in the lungs of the Balb/c mice (n = 5) was determined by plaque assay on Vero cells. The data represent the mean ± SD of three independent experiments, each performed in duplicates. The results are expressed as relative ratios of plaque numbers in MHV-68 infected mice primed with siRNA, (NS)siRNA, or PBS treated groups to that in the MHV-68 infected group (virus control, 100%). Bars (**A**–**C**) represent average ± s.d. of five mice. Columns with different alphabets indicate the values to be statistically significant (p < 0.05) by LSD.

MHV-68 is regarded as a substitute for human *γ-herpesvirus* and has been widely used in *in vivo* studies^15^. We used mouse siRNA specific to IFITM1 to appreciate the effect of IFITM1 on MHV-68 infection, *in vivo*, using BALB/c mice. The experimental design is detailed in the schematic (Fig. 4). Priming mice with siRNA specific to IFITM1 significantly lowered expression of IFITM1 in the lungs of the mice compared to (NS)siRNA (Fig. 5A). Our data demonstrates a significant increase in the leukocyte count in mice that were infected with MHV-68 compared to the uninfected mice (Table 1). The leukocyte count in infected mice that were primed with siRNA specific to IFITM1 was significantly lower compared to those that were primed with PBS or (NS)siRNA. We did not notice atypical lymphoid monocytes in these animals.

**Table 1:** Leukocyte count in the experimental groups.

Groups (n = 5)	Number of leukocytes per 1 µl of blood
Uninfected	11633 ± 1500
MHV-68	14480 ± 1943
siRNA + MHV-68	11031 ± 2179
(NS)siRNA + MHV-68	14520 ± 2431
PBS + MHV-68	14729 ± 1735

MHV-68 infection of mice was monitored by the traditional plaque assay. Plaque assay could detect MHV-68 in lungs of the infected mice (Fig. 5B). MHV-68 infection was significantly inhibited in the mice that were primed with siRNA specific to IFITM1 compared to those that were primed with (NS)siRNA or PBS (Fig. 5B). The results were confirmed by performing qRT-PCR (Fig. 5C). Taken together, silencing the expression of IFITM1 significantly hampered MHV-68 infection of mice.

## Discussion

Many different types of cellular sensors can detect viruses and induce the expression of the type I interferons (IFNs)—IFN-α and IFN-β. Type I IFNs bind to the ubiquitously expressed IFNAR (IFN-α/β receptor), activating the JAK/STAT pathway^17,18^. The type I IFN response can induce the expression of hundreds of interferon stimulated genes (ISGs) which primarily serve to limit further virus spread and infection^19^. IFITMs are a family of proteins that have gained popularity as novel antiviral ISGs^20^. IFITMs are a member of the interferon-induced 125–133 aa protein family including IFITM1, IFITM2, IFITM3, IFITM5, and IFITM10. This family of proteins is located on chromosome 11 of the human genome and originally described as highly inducible genes by α- and γ-interferons (IFNs)^21,22^. IFITM proteins are significantly upregulated by type I and II IFNs and are critical for anti-viral innate immune responses^23,24^. Recent reports indicate IFITMs play a significant role in virus entry. IFITMs inhibits entry of many RNA viruses including influenza A H1N1 Virus, West Nile Virus, Dengue Virus, HIV, and HCV^23,25,26^. However, IFITMs can also enhance viral infection of cells: (i) both IFITM1 and IFITM3 modestly enhance human papillomavirus 16 (HPV-16) infection of a variety of cells;^13^ (ii) Zhao *et al*. have shown type I IFN-α, IFN-γ, and type II IFN-λ to significantly promote infection of human coronavirus, HCoV-OC43 by the induction of IFITM proteins. The authors reported that the over-expression of IFITM3 significantly increased susceptibility of Huh7.5 cells to HCoV-OC43 infection;^14^ and (iii) Inhibition of IFITM1 expression by specific miR-36 mimic significantly inhibited early stages of KSHV infection of human B and endothelial cells^10^. In general, the IFITM family of proteins affects virus entry of cells.

In the current study, we demonstrated that EBV, another *γ-herpesvirus*, induces IFITM1 expression during the early stages of infection and it followed an identical pattern as that of KSHV (Fig. 1A,B). Binding of KSHV and EBV to target cells is not sufficient to induce IFITM1 expression (Fig. 1C). We went one step further in demonstrating that over-expressing IFITM1 significantly alters not only the internalization of the *γ-herpesviruses* (Fig. 2A) but also the ability to establish infection as monitored by the expression of the respective viral transcripts (Fig. 2B). Also, silencing expression of IFITM1 by specific siRNAs resulted in a significant drop in the number of internalized viral particles (Fig. 2E). IFITM1 promotes *γ-herpesvirus* infections at a post-binding stage of entry as silencing IFITM1 did not alter the KSHV and EBV binding to the cell surfaces (Fig. 3). KSHV utilize HS as binding receptor^6^. EBV also interacts with HS expressed on epithelial cells via glycoprotein gp150^27^. Generally, speaking, BJAB cells are thought to be resistant to KSHV and EBV primarily because B cells do not express HS^28^. Recent studies determined BJAB cells to express HS^28^ and that KSHV and EBV can efficiently infect them^6,29,30^. In our hands, treating EBV with soluble heparin blocked binding to BJAB cells due to one or both of the following reasons: (i) Just like MHV-68 and KSHV, HS may have a role to play in EBV infection of BJAB cells; and (ii) High concentrations of soluble heparin used could have a steric bulk effect on the virus binding to cells. We are aware of this discrepancy associated with the role of HS in EBV binding and entry. In the current study, soluble heparin was only used as a control and further elaborate studies should be conducted prior to ruling in or out a role for HS in EBV entry into BJAB cells.

A natural model system to investigate *γ-herpesvirus*-host interactions is the infection of mice with MHV-68, a natural pathogen of wild rodents^31,32^. Therefore, to understand the *in vivo* effects of IFITM1 on *γ-herpesviruses*, we used MHV-68 as a model virus. Leukocyte counts were increased in response to MHV-68 infection of mice (Table 1). This increase in the leukocyte count was significantly lowered when the mice were primed with siRNA compared to (NS)siRNA and PBS. We did not observe atypical lymphoid monocytes in the mice from all the groups used as it was too early to observe any such changes. Priming mice with siRNA specific to IFITM1 significantly lowered expression of IFITM1 in lungs compared to PBS, (NS)siRNA (Fig. 5A). It was determined by plaque assay and qPCR that a decrease in the expression of IFITM1 resulted in a significant decrease in MHV-68 infection of lungs obtained from the mice (Fig. 5B,C). This study focused primarily in monitoring infection of lungs because intranasal MHV-68 infection of mice results in acute lytic infection of lungs followed by the establishment of lifelong latency^33^. Taken together, we demonstrate for the first time a crucial role for IFITM1 in the *in vivo* infection of *γ-herpesviruses*.

The results from this study put forth crucial questions in terms of better understanding the role of IFITM1 in altering entry of *γ-herpesviruses*. Numerous mechanisms by which IFITM proteins alter virus entry have been proposed and it is not limited to the following: (i) acting as pattern recognition receptors by sensing virus infection and activation of downstream cellular signaling pathways;^23^ and (ii) reducing membrane fluidity and curvature, and by possibly disrupting intracellular cholesterol homeostasis^34,35^. Albeit, none of the mechanisms have been confirmed. Future studies will be aimed at delineating that eluding mechanism by which IFITM1 enhance the internalization of the *γ-herpesviruses*. These studies are crucial because they help us understand the role of ISGs in modulating *γ-herpesvirus* infection of cells. In addition, the results from this study will benefit in designing IFITM1 targeted therapies to treat cancers^36,37^, and *γ-herpesvirus* infections including that of HIV^38^ and HCV^39^.

## Materials and Methods

### Cells

Human Burkitt lymphoma B cell line (BJAB) was used in this study. BJAB cells were propagated in phenol red–free RPMI medium (Invitrogen, Carlsbad, CA) containing 10% charcoal-stripped fetal bovine serum (FBS; Atlanta Biologicals, Lawrenceville, GA), L-glutamine, and antibiotics^40^. The cells used in this study were negative for mycoplasma as tested by Mycoplasma PCR ELISA (Roche Life Science, Indianapolis, IN).

### Virus and mice

The viruses used in this study were wild-type KSHV^41^, EBV^42^, and MHV-68^43^. Female 6-week-old inbred BALB/c mice supplied by the Faculty of Veterinary Medicine, Brno, Czech Republic were used in this study. We generated ultraviolet (UV) inactivated KSHV (UV.KSHV) and EBV (UV.EBV) as per early studies^10^.

### Virus infection of cells, RNA and DNA extraction, and monitoring virus infection

BJAB cells were infected with 10 multiplicity of infection (MOI) of KSHV and EBV. The cells were left uninfected or infected for 5, 10, 15, and 30 min prior to washing the cells twice in PBS and processed appropriately for RNA and DNA extraction. Total RNA was extracted using TRIzol (Invitrogen, Carlsbad, CA). The RNA concentration was measured with a NanoDrop ND-2000 spectrophotometer (Thermo Fisher Scientific, Waltham, MA), and then verified for quality using an Agilent 2100 Bioanalyzer (Agilent Technologies, Santa Clara, CA). Only the RNA samples with 260/280 ratios of 1.8 to 2.0 were used in the study. Viral DNA was extracted by isolating total genomic DNA and determining the internalized virus particles by qPCR^44^.

Extracted RNA was used to synthesize cDNA and the expression of *ORF50* was monitored by qRT-PCR using specific primers as per earlier studies^41^. Expression of *ORF50* was used as a scale to measure KSHV infection of cells. As reported earlier^45^, the lowest limit of detection in the standard samples was 6–60 copies of the *ORF50* gene. The results from the use of *ORF50* primers were consistently confirmed by monitoring the expression of another viral immediate early (IE) gene, vGPCR (data not shown). EBV infection was monitored using specific primers to *BRLF1* (homolog of KSHV *ORF50*)^46^.

### Transfection of cDNA

Target cells were transiently transfected with plasmid DNA using FuGene HD (Promega, Madison, WI) as per manufacturer’s recommendations. The plasmid, pQCXIP encoding IFITM1, used in this study was kindly gifted to us by Dr. Michael Farzan (The Scripps Research Institute, Jupiter, USA). FuGene HD/DNA ratios of 3:1 for adherent cell lines and 6:1 for suspension cell lines were used. These were transient transfections and experiments using these cells were conducted 48 h post transfection.

### *In vitro* silencing IFITM1 using siRNA

Expression of IFITM1 was inhibited by the transfection of double-stranded (ds) RNA oligos as per standard protocols^10^. IFITM1 siRNA was purchased from Dharmacon RNA Technologies (Lafayette, CO). Briefly, untransfected cells and cells transfected with siRNA or (NS)siRNA for 12 h were infected with 10 MOI of KSHV. At the end of 30 min PI, KSHV infection was assessed by monitoring *ORF50* expression by qRT-PCR.

### Binding assay

The effect of IFITM1 on KSHV binding to target cells was assessed by PCR detecting the cell-bound KSHV DNA. Briefly, untransfected cells or cells transfected with (NS)siRNA, or siRNA specific to IFITM1 were infected with 10MOI of KSHV at +4 °C. After 60 min of incubation with virus, cells were washed three times with PBS to remove the unbound virus. Cells were pelleted, and total DNA including those representing the cell bound KSHV was isolated using DNeasy kit (Qiagen, Valencia, CA) and subjected to qPCR analysis monitoring *ORF50* according to recently published work^45^. Incubating KSHV with 500 µg/ml of heparin and chondroitin sulfate A (CSA; Sigma-Aldridge) for 1 h at 37 °C were used as known positive and negative controls.

### Electron microscopy

EM studies were conducted to appreciate the effect of IFITM1 on virus binding to cells. Briefly, viruses were allowed to bind at 4 °C to BJAB cells that were untransfected, or transiently transfected with IFITM1-specific siRNA, or (NS) siRNA. After 60 min cells were fixed with 2% glutaraldehyde. Thin sections were examined by transmission electron microscopy. The number of virus particles adsorbed on the cell membranes were counted as per procedures outlined in earlier studies^47^.

### Animal studies

Animal studies using BALB/c mice was performed to understand the effects of IFITM1 on γ-herpesviruses, *in vivo*. Briefly, five groups of mice (n = 5/group) used in this study were (i) virus free or uninfected; (ii) MHV-68 infected; (iii) IFITM1 siRNA primed + MHV-68 infected; (iv) (NS)siRNA primed + MHV-68 infected; and (v) PBS primed + MHV-68 infected. Mice were primed with PBS, 20 µg/mouse of IFITM1-siRNA, or (NS)siRNA on day 0 by tail vein injection. The uninfected and the MHV-68 infected groups were left untreated. On day 02, only groups (iii), (iv), and (v) were tail vein-injected with IFITM1 siRNA, (NS)siRNA, and PBS, respectively. After this step, animals in groups (ii), (iii), (iv), and (v) were intranasally (i.n) infected with 2 × 10^4^ PFU (20 µl) of MHV-68. On day 04, the animals in groups (iii), (iv), and (v) received tail vein injections of IFITM1 siRNA, (NS)siRNA, and PBS, respectively; while animals in groups (i) and (ii) were left undisturbed. On day 06, mice were sacrificed and lungs were collected. Relative IFITM1 gene expression was calculated using the ${2}^{-\triangle \triangle {C}_{T}}$ method^48^, using β-actin mRNA expression as reference gene and the virus free group as the calibrator. Plaque assay^49^ and qPCR^50^ was conducted to determine MHV-68 infection of lungs.

All animal experiments were performed according to the European Union standards, and fundamental ethical principles including animal welfare requirements were respected. All experiments were done with the approval of State Veterinary and Food Administration of the Slovak Republic (2937/10221).

### Ethical statement

All animal experiments were performed according to the European Union standards, and fundamental ethical principles including animal welfare requirements were respected. All experiments were done with the approval of State Veterinary and Food Administration of the Slovak Republic (2937/10221).

## Electronic supplementary material

Supplemental data
