# Supplementary material for: IFITM1 expression is crucial to gammaherpesvirus infection, in vivo
Source: Sci Rep. 2018 Sep 20;8:14105. doi: 10.1038/s41598-018-32350-0 (PMC6149222; doi:10.1038/s41598-018-32350-0)
Supplement: Supplementary file 1 — Supplemental data [file 41598_2018_32350_MOESM1_ESM.docx]

### **IFITM1 expression is crucial to** [***gammaherpesvirus* infection, *in vivo***](https://www.ncbi.nlm.nih.gov/pubmed/12879740)

**Hosni A.M. Hussein^1€^, Katarina Briestenska^2,3€^, Jela Mistrikova^2,3^, Shaw M. Akula^1^***

^1^Department of Microbiology & Immunology, Brody School of Medicine at East Carolina University, Greenville, NC 27834; ^2^Department of Microbiology and Virology, Faculty of Natural Sciences, Comenius University in Bratislava, Mlynská dolina, SK-842 15 Bratislava, Slovak Republic; ^3^Institute of Virology, Biomedical research Center, Slovak Academy of Sciences, Dubravska cesta 9, 845 05 Bratislava, Slovak Republic.

**Short title:** IFITM1 enhances *gammaherpesvirus* infection

**Word count for text:** 3118

**Word count for abstract:** 182

**Figure:** 5

**Table:** 1

**Reference count:** 50

**Supplemental Info:** Supplemental figures 1-6

^€^These authors contributed equally to this work.

*Corresponding Author: Shaw M. Akula, Department of Microbiology & Immunology, Brody School of Medicine, East Carolina University, Greenville, North Carolina, USA 27834. Phone: (252)744-2702; Fax: (252) 744-3104; Email: [akulas@ecu.edu](mailto:akulas@ecu.edu)

**Supplemental Fig. 1: Infection of HMVEC-d cells with EBV and KSHV induce expression of IFITM1.** The relative expression of IFITM1 in EBV or KSHV infected HMVEC-d cell was monitored by qRT-PCR. The expression was measured in terms of cycle threshold value (Ct) and normalized to expression of β-actin. The *x-axis* denotes the time point post virus infection in minutes and the *y-axis* denotes fold change in expression of IFITM1. Bars represent average ± s.d. of five individual experiments. Columns with different alphabets indicate the values to be statistically significant (p < 0.05) by LSD.


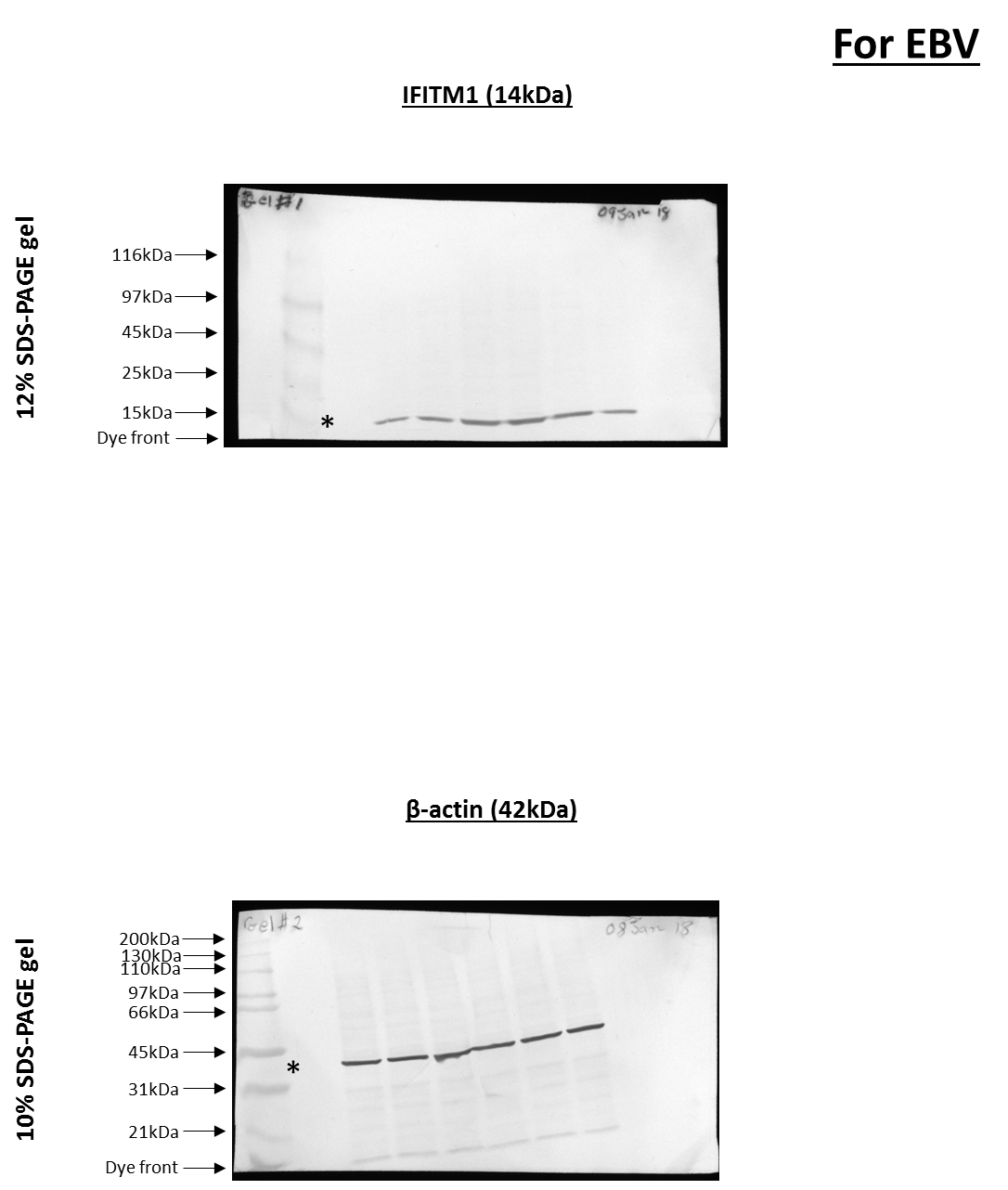


**Supplemental Figure 2.** Original Western blots of the data presented in Figure 2 (EBV)


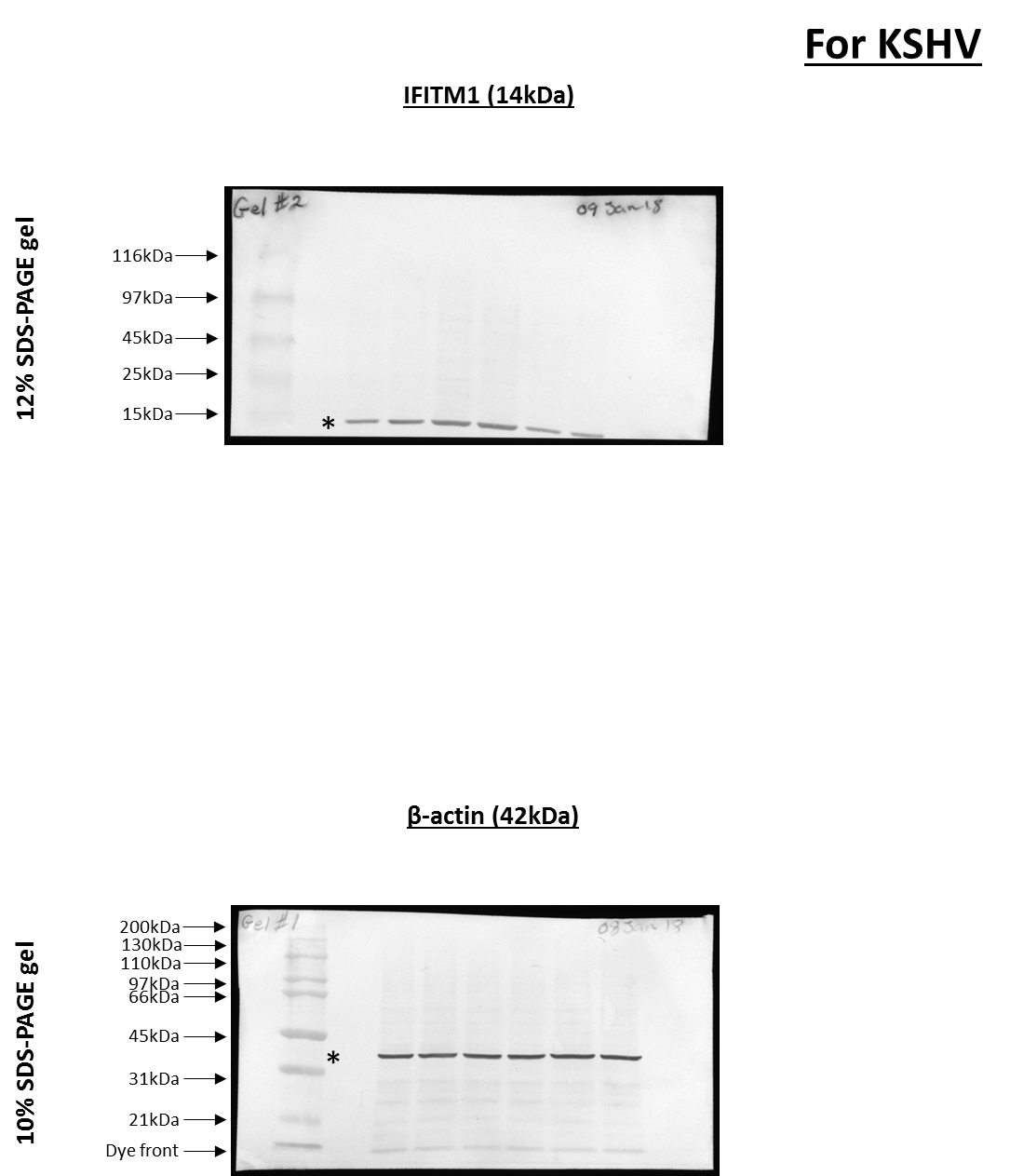


**Supplemental Figure 3.** Original Western blots of the data presented in Figure 2.

**Supplemental Figure 4:** Original Northern blots of the data presented in Figure 3C.

**Supplemental Figure 5:** Original Western blots of the data presented in Figure 3D. The circle denotes air bubble.

**Supplemental Fig. 6**: **IFITM1 enhancement of EBV and KSHV infection of cells is at a post-attachment stage of virus entry.** EBV and KSHV binding to BJAB cells were monitored in cells that were untransfected, untransfected and treated with heparin or CSA, or transiently transfected with IFITM1-specific siRNA, or transfected with non-specific (NS) siRNA. A MOI of 1 was used in this study. Data was plotted to represent the percentage of EBV or KSHV binding to BJAB cells treated differently compared to the untransfected cells. Bars represent average ± s.d. of five individual experiments. Columns with different alphabets indicate the values to be statistically significant (p < 0.05) by LSD.
